# Pediatric Sleep Surgery

**DOI:** 10.3389/fped.2014.00051

**Published:** 2014-06-05

**Authors:** Cecille G. Sulman

**Affiliations:** ^1^Department of Otolaryngology and Communication Sciences, Medical College of Wisconsin, Milwaukee, WI, USA

**Keywords:** obstructive sleep apnea in children, tonsillectomy, expansion pharyngoplasty, craniofacial distraction, distraction osteoneogenesis, tongue base surgery, tracheostomy

## Abstract

Adenotonsillectomy is the most common surgery performed for sleep disordered breathing with good outcomes. Children with obesity, craniofacial disorders, and neurologic impairment are at risk for persistent sleep apnea after adenotonsillectomy. Techniques exist to address obstructive lesions of the palate, tongue base, or craniofacial skeleton in children with persistent sleep apnea. Children with obstructive sleep apnea have a higher rate of peri-operative complications.

## Introduction

The prevalence of snoring has been reported in 3–12% in children (1) although some studies suggest that the rate may be as high as 27% (2). Approximately 40% of children who snore have more significant manifestations with obstructive sleep apnea syndrome (OSAs). Major pre-disposing factors for upper airway obstruction (UAO) include anatomic narrowing, abnormal mechanical linkage between airway dilating muscles and airway walls, muscle weakness, and abnormal neural regulation (3). Complications related to severe OSAs include cor pulmonale, right ventricular hypertrophy, congestive heart failure, alveolar hypoventilation, pulmonary hypertension, pulmonary edema, and failure to thrive (4). There is also risk for permanent neurological damage and even death (4). The primary surgical approach in children with clear symptoms of sleep disordered breathing (SDB) and tonsillar hypertrophy is adenotonsillectomy. This review explores this technique in addition to others that address other sites of UAO.

## Evaluation of the Patient with Sleep Disordered Breathing

### History

Sleep disordered breathing and UAO are clinical diagnoses related to snoring, breath-holding, labored breathing, and gasping while sleeping (Table 1) (5, 6). OSAs is a diagnosis based on sleep study findings. Populations at risk include those with obesity (7, 8), Down syndrome, craniofacial syndromes (9–11), mucopolysaccharide disease, and the neurologically impaired child. A careful history is performed to ensure sleep surgery is warranted due to the significant morbidity involved with upper airway surgery.

**Table 1:** **Symptoms of sleep disordered breathing**.

Snoring	Breath-holding/pausing
Gasping	Choking
Mouth breathing	Enuresis
Sleepwalking	Night terrors
Sleep talking	Bruxism
Morning headache	Halitosis
Behavioral/neurocognitive disorders	Dysphagia

### Physical examination

A general appearance of failure to thrive or obesity in the setting of a history of SDB is concerning. An open mouth posture and stertorous or sonorous breathing may indicate upper airway resistance. Craniofacial abnormalities may include midface hypoplasia or mandibular hypoplasia as in Crouzon syndrome or Pierre Robin sequence. Anterior rhinoscopy may reveal edematous mucosa, inferior turbinate hypertrophy, or septal deviation. Findings in the oropharynx contributory to obstructive breathing include tonsillar hypertrophy, macroglossia, palatal narrowing, and elongation. Airway evaluation with a flexible nasopharyngoscope provides additional information regarding anatomic sites of obstruction including septal deviation, adenoid hypertrophy, retropalatal or retrolingual narrowing, enlarged tongue base, lingual tonsil hypertrophy, or supraglottic collapse (12). Endoscopy in a drug-induced sleep state with dexmedetomidine further highlights obstructive lesions that may not be revealed during an awake flexible laryngoscopy.

### Additional evaluation

History alone may not be sufficient in diagnosing pathologic SDB. Polysomnogram (PSG) is the gold standard for diagnosis (13) and should be considered in children with complex medical conditions such as obesity, Down syndrome, craniofacial abnormalities, neuromuscular disorders, sickle cell disease, or mucopolysaccharidoses (14). PSG is also indicated in children for whom the need for surgery is uncertain, such as in the case of tonsillar hypertrophy and lack of sufficient history, or when significant symptoms are present in the face of a negative physical examination (15). Results of the study should be relayed to the anesthesia team prior to surgery.

High kilovoltage lateral neck imaging may be used to evaluate for adenotonsillar hypertrophy in a child who has a difficult examination or a caregiver defers flexible endoscopy (16). Chest radiography should be ordered to look for evidence of pulmonary hypertension or right ventricular hypertrophy in a child with severe obstructive sleep apnea (17). Cine magnetic resonance imaging is a useful radiographic adjunct to the physical examination because it allows the clinician to screen for and to observe airway collapse in three planes (axial, coronal, and sagittal) (18, 19). This is particularly helpful in isolating anatomic sites of airway obstruction in children who have persistent apnea after adenotonsillectomy. Patients with severe obstructive sleep apnea or abnormal electrocardiogram findings on PSG warrant an echocardiogram; On occasion, a normal electrocardiogram may occur even with echocardiographic findings of pulmonary hypertension (17). Laboratories are not routinely ordered prior to surgical intervention unless history is concerning for a bleeding disorder (20).

## Medical Intervention

Detailed discussion of medical intervention is beyond the scope of this article; medical therapy will be briefly addressed. Antibiotics are not beneficial in long-term reduction of tonsil hypertrophy, although during acute inflammation, broad spectrum antibiotics may provide a short term decrease in tonsil size; one study reported only 15% of patients avoided surgery in long-term follow up (21). Intranasal steroids are helpful in mild OSA if adenoid hypertrophy is the pre-dominant etiology (22–24). If residual sleep apnea (AHI 1–5) is present after adenotonsillectomy, the use of intranasal steroid and montelukast has been demonstrated to improved and/or normalized respiratory and sleep disturbances (25). Oral appliances or functional orthopedic appliances may be helpful in the treatment of children with craniofacial anomalies that are risk factors of apnea (26, 27).

## Surgical Techniques

### Adenotonsillectomy

Adenotonsillectomy alone is an effective and durable treatment for most children (28) and is the first choice for children with OSA (28–30). Adenotonsillectomy may be performed safely in an outpatient setting in children greater than 3 years of age with an American Society for Anesthesia (ASA) Class I/II (low anesthesia risk) (31). The post-operative complication rate is higher in children with craniofacial disorders, failure to thrive, neurological impairment, Down syndrome, obstructive sleep apnea, and children age 3 years or less (32). Children who are at risk for peri-operative complications should remain post-operatively for observation.

Multiple techniques and devices may be used to perform tonsillectomy, which may be influenced by surgeon exposure in training programs and operator experience. Patient recovery times, post-operative morbidities, as well as cost related to the device are also important factors that may drive device choice. Consensus has not been reached regarding the optimal technique with the lowest morbidity (33, 34).

Most commonly, techniques used include cold steel dissection and electrosurgical dissection. During cold steel dissection, the tonsil is grasped, and after identification of the tonsil capsule, the tonsil is snared and removed. Electrocautery is typically used for hemostasis. Electrosurgical dissection uses thermal energy to dissect tissues with either a monopolar or bipolar tip with minimal blood loss (35). Post-operative morbidities for these techniques are similar, and include hemorrhage (1.2–2.1%), dysphagia, and otalgia (35). Plasma surgical dissection ablates and coagulates soft tissue by generating an ionized plasma layer with a radiofrequency current that breaks molecular bonds, producing a melting tissue effect. Many studies have shown this technique to cause less pain, shorten the recovery period and require less post-operative narcotics than other methods of tonsillectomy (36). The Harmonic scalpel (vessel sealing system) uses ultrasonic energy to vibrate its blade, providing simultaneous cutting, and coagulation of the tissue (37). Post-operative hemorrhage and intraoperative blood loss is low with the Harmonic scalpel, however, operative times are longer compared to other techniques (38). Studies are conflicted on post-tonsillectomy pain outcomes (37).

Partial tonsillectomy, also known as tonsillotomy or intracapsular tonsillectomy, may be performed for tonsil hypertrophy with SDB/OSAs, but is contraindicated for chronic tonsillitis. The premise of partial tonsillectomy is reduction of obstructive tonsillar tissue while sparing the tonsillar capsule, thus preventing exposure of the underlying pharyngeal muscles and decreasing post-operative pain. Technology utilized for partial tonsillectomy includes a microdebrider or radiofrequency device. An advantage of partial tonsillectomy includes decreased post-operative hemorrhage rates, however, intraoperative bleeding is increased, and may impede visualization at times (39). Studies on the long-term efficacy of partial tonsillectomy compared to established approaches are limited, but promising (40).

Adenoidectomy may also be performed with a variety of devices, primarily through a transoral approach. Devices include dissection with an adenoid curette, suction monopolar cautery, microdebrider, coblation, and plasma devices. Revision adenoidectomy occurs at a rate of 1.3% (41). Reasons for revision include persistent symptoms with adenoid re-hypertrophy, chronic adenoiditis, and recurrent otitis.

The most common complications of adenotonsillectomy are related to anesthesia risks and breathing concerns, pain, otalgia, and bleeding (42). Dehydration may occur due to poorly controlled pain, refusal of oral intake, nausea, and vomiting secondary to narcotic use (42). Rare complications of tonsillectomy include subcutaneous emphysema, pneumomediastinum, and taste disturbance due to damage to the lingual branch of the glossopharygngeal nerve (43, 44). Nasopharyngeal stenosis, also an uncommon outcome, results from approximation of raw mucosal surfaces during the healing process (29).

Success rates for adenotonsillectomy range from 59.8 to 85%, with a significant improvement in AHI from pre-operative levels (29) as well as an overall improvement in quality of life (45–47). Studies continue to evaluate functional outcomes after adenotonsillectomy. Of note, children with severe pre-operative OSA, asthma, age greater than 7, or obesity are at risk for persistence of OSA after adenotonsillectomy (48–50). Adenotonsillectomy outcomes may not be favorable in patients with severe pre-operative OSA or when obesity is present (51, 52), however, it remains the currently recommended initial treatment for OSA in obese patients. Increasing rates of residual obstructive sleep apnea indicate a future role for adjunct medical as well as surgical interventions. Post-operative PSGs are recommended 6–8 weeks following surgery for those with additional risk factors for OSA, including age younger than 3 years, severe OSA present on pre-operative PSG, cardiac complications of OSA (e.g., right ventricular hypertrophy), failure to thrive, obesity, pre-maturity, recent respiratory infection, craniofacial anomalies, and neuromuscular disorders (Table 2) (53). Patients with mild-to-moderate OSAS who have complete resolution of signs and symptoms do not require repeat PSG (54). Post-operative reports of symptoms such as snoring and witnessed apneas correlate well with persistence of OSA after adenotonsillectomy (55).

**Table 2:** **Indications for post-operative PSG**.

Age <3	Severe pre-operative PSG
Cardiac complications of OSA	Failure to thrive
Craniofacial anomalies	Neuromuscular disorders
Obesity	Persistent SDB

### Surgery for multilevel obstruction

Multilevel obstruction involving any combination of the nasal, nasopharyngeal, retropalatal, retroglossal, and hypopharyngeal anatomy may be found in otherwise healthy children. However, certain populations are pre-disposed to multilevel airway collapse, including children who have obesity, nasal obstruction, neurological impairments, laryngotracheomalacia, laryngotracheal or bronchial stenosis, and craniofacial anomalies, such as Pierre Robin sequence and Down syndrome (55). The exact nature of dynamic airway collapse may not be appreciated by a detailed history and physical examination, therefore cine MR imaging and flexible endoscopy performed in the office and the operating room may help identify the anatomic level(s) of obstruction (14).

There is limited evidence available in children regarding surgical techniques for multilevel OSA. Lack of objective PSG criteria for comparison between surgical treatment options as well as the nature of surgical intervention makes it difficult to conduct blinded studies (38). A staged approach should be considered in children; Prager et al. (56) described an 8.2% incidence of oropharyngeal scarring and stenosis in 48 children who underwent multilevel surgery that included lingual tonsillectomy for OSA in children. Even solitary surgical improvements in airway size can augment airway dynamics and reduce the Bernoulli and Starling effects and collapse at other levels (57).

#### Nasal surgery

A deviated septum or turbinate hypertrophy may be contributory to nasal airway obstruction. Septoplasty should be performed with careful patient selection utilizing a limited approach. Complications include persistent septal deviation, bleeding, and septal perforation. Septoplasty may be useful in improving continuous positive airway pressure (CPAP) tolerance, particularly in older children.

Two main techniques exist for turbinate volume reduction: radiofrequency ablation and microdebrider-assisted reduction. A review of turbinate surgery in children comparing both techniques for nasal obstruction demonstrated that both are effective (58), however, maintenance of improvement at 3 years was better with the microdebrider-assisted technique (59). Mild-to-moderate edema with subsequent nasal obstruction and thick mucus formation can be expected for the first week after the procedure (57). If mucosal erosion is present, the risk of post-operative bleeding and adherent crust formation increases with radiofrequency ablation (59, 60).

#### Oropharyngeal surgery

Patients with an elongated palate may benefit from techniques to widen or stabilize the palate, including lateral pharyngoplasty, expansion sphincter pharyngoplasty, and uvulopalatopharyngoplasty (UPPP). Palatal procedures may be performed with and without adenotonsillectomy. Outcomes are better known for adults who have a higher frequency of undergoing palatal reduction techniques.

**Figure 1:**
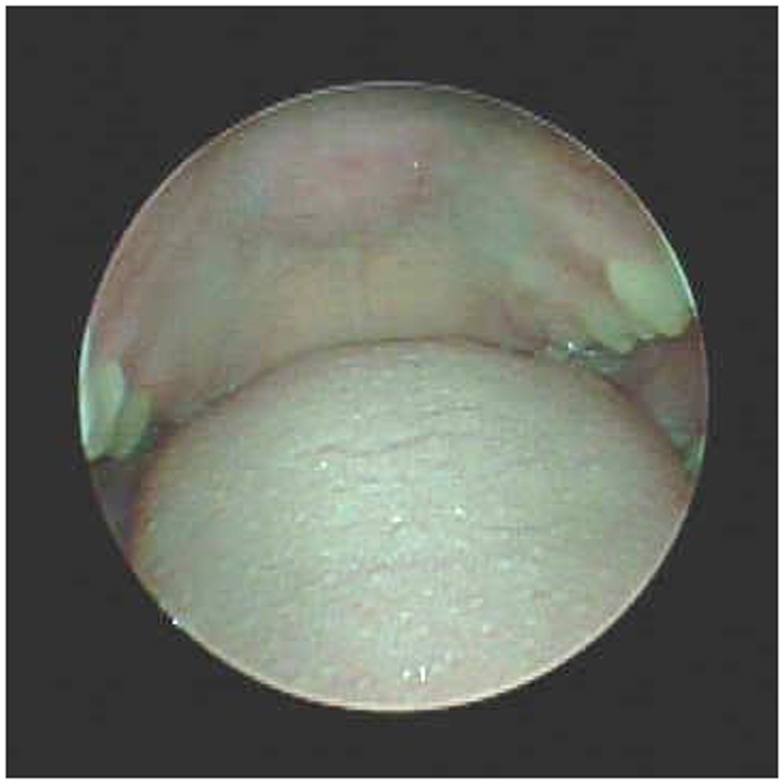
**Pre-operative view of palate, Mallampati 4**.

**Figure 2:**
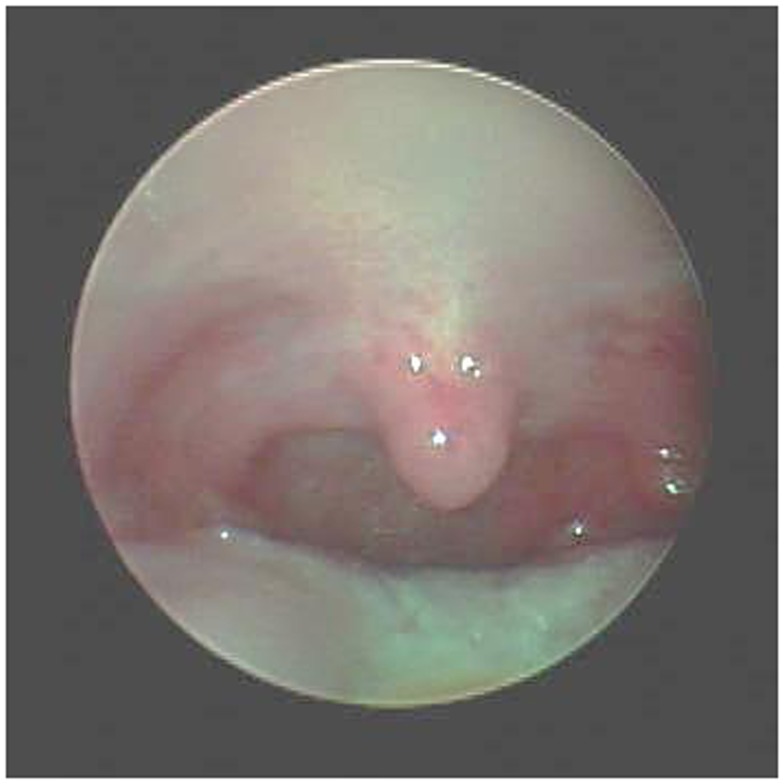
**Post-operative view of palate after lateral sphincter pharyngoplasty**.

Lateral pharyngoplasty is a minimally invasive procedure, which involves suturing the tonsillar pillars together over the tonsillar fossa after the tonsils are removed, reducing the collapsibility of the pharynx (61). Expansion sphincter pharyngoplasty is a more technically involved approach, with rotation and suspension of the palatopharyngeus muscle onto the soft palate, sparing the uvula. This technique provides stabilization of the palate in addition to an improved diameter of the oral airway (Figures 1 and 2). This may be incorporated into the initial surgical approach with tonsillectomy or as a secondary procedure in patients who have persistent sleep apnea after adenotonsillectomy. Pang and Woodson demonstrated an improvement in AHI from 44.2 ± 10.2 to 12.0 ± 6.6 (*p* < 0.005) following expansion sphincter pharyngoplasty in select patients with small tonsils and an elongated palate (62). Long-term outcomes in children for both lateral pharyngoplasty and expansion sphincter pharyngoplasty are yet to be determined.

Uvulopalatopharyngoplasty involves removal of the soft palate and uvula, widening the oropharyngeal airway. UPPP has been reported to be successful in children with cerebral palsy and hypotonic upper airway muscles (63). Although an initial report of UPPP in an otherwise normal child was promising (64), UPPP is not commonly performed in children. Significant complications such as nasopharyngeal stenosis, palatal incompetence, and speech difficulties may occur with this technique (65).

#### Tongue base surgery

**Figure 3:**
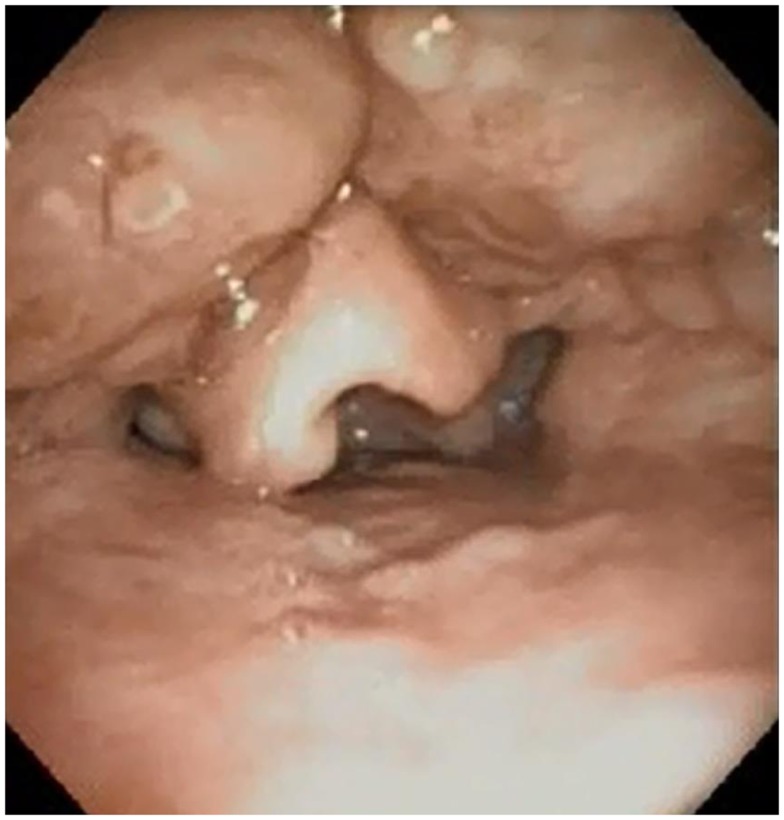
**Lingual tonsil hypertrophy visualized on sleep endoscopy**.

Surgical procedures to increase the volume of the retrolingual airway include lingual tonsillectomy, glossectomy, and advancement and suspension procedures. Figure 3 demonstrates lingual tonsil hypertrophy in a child who presented with obstructive sleep apnea after adenotonsillectomy. Lingual tonsillectomy may be performed with sharp dissection, monopolar dithermy (66), laser (67), or coblation (12, 54). The anterior midline of the tongue is controlled with a heavy silk suture, while an appropriately sized laryngoscope is used to expose the tongue base during lingual tonsillectomy (12). Lin performed coblation lingual tonsillectomies on 26 patients (aged 3–20 years) with polysomnography-proven persistent obstructive sleep apnea after adenotonsillectomy, as well as a diagnosis of lingual tonsillar hypertrophy made by flexible fiberoptic sleep endoscopy. Comparison of pre-operative and post-operative PSG demonstrated statistically significant reductions in the respiratory distress index (12). Complications related to lingual tonsillectomy include edema (67) and adhesions between the epiglottis and tongue base (68).

Glossectomy or tissue volume reduction procedures decrease tongue volume and proportionally increase airway size. Glossectomy involving a wedge of tongue muscle is an effective technique (12) that may be applied to children with significant tongue hypertrophy as in Beckwith–Wiedemann or Down syndrome. Midline glossectomy is performed open or via a minimally invasive technique submucosally (12). Success rates for the submucosal minimally invasive lingual excision are reported to be 60% (12). Risks include airway edema, hematoma, abscess formation, and permanent hypoglossal injury (54).

Genioglossus advancement to advance the tongue requires a midline osteotomy of the mandible (69), however, this technique is not amenable in small children due to the presence of tooth buds. In combination with radiofrequency ablation of the tongue, patients have demonstrated improvement in the AHI, with lower success rate in children with Down syndrome (14). A minimally invasive approach (Repose) involves passing of a suture through the tongue base, which is then stabilized to the medial aspect of the mandible with a screw (70). Post-operative complications include wound infection, edema, and seromas. Asymptomatic tissue anchor barb fractures have been noted (71). Long-term effectiveness is unknown at this time.

Radiofrequency ablation described by Powell et al. (72) involves insertion of a two-pronged probe generating thermal damage at multiple points in the tongue base. Tongue bulk and flaccidity of the tongue base is reduced through fibrosis (14). A low rate of complications (3.4%) is reported, ranging from mucosal ulceration, to superficial infection, and transient paresthesia of the hypoglossal nerve (73).

#### Laryngeal surgery

**Figure 4:**
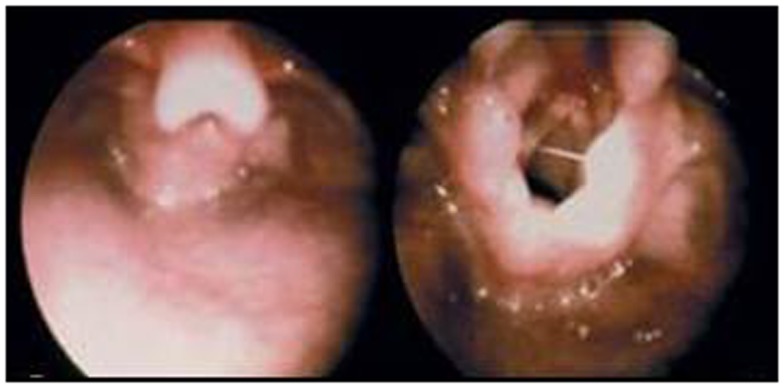
**Left, severe laryngomalacia with epiglottic collapse with inspiration; Right, tight aryepiglottic folds and redundant arytenoid mucosa with inspiration**.

Laryngomalacia, or obstruction of the glottis secondary to collapse of supraglottic structures, is diagnosed with flexible laryngoscopy. This condition is primarily seen in infancy, but may present in older children (Figure 4). For the majority of patients, laryngomalacia may be managed expectantly without intervention. In the setting of OSA, failure to thrive, or feeding difficulties, a supraglottoplasty is indicated. Sharp dissection or laser techniques are used to reduce redundant mucosa or incise shortened aryepiglottic folds (74). Medical comorbidities are associated with worsened post-operative outcomes, although the majority of children improve after supraglottoplasty (75).

#### Craniofacial surgery

Distraction osteogenesis (DOG) is a surgical technique used to expand the facial skeleton in children with congenital micrognathia or midface hypoplasia without the need for bone grafting (76) and is effective in treating craniofacial deformities (77, 78). In combination with adenotonsillectomy, maxillary distraction has a cure rate of 87.5% in children with sleep apnea (79). Additionally, this technique may avert the need for tracheostomy in children with Pierre Robin sequence, but is less successful in children with complex congenital syndromes (77). Complications include pre-mature callus consolidation requiring another DOG procedure, cheek abscess requiring incision and drainage, minor lip erosion from pin contact, facial cellulitis, unilateral facial paralysis, and temporal mandibular joint ankylosis (77).

Rapid maxillary expansion (RME) involves the use of an oral appliance that is adjusted daily to increase palatal width. RME presents another option of treatment for children with high-arched palates with associated increased nasal resistance and mild OSAs (80). A high-arched palate may also be associated with a posterior tongue posture, which contributes to retroglossal airway narrowing (81). RME is most effective in pre-pubertal children prior to palatal suture closure. RME may be used in combination with adenotonsillectomy to improve the nasal and oral airway (82).

#### Tracheostomy

Patients who are refractory to surgical therapy or CPAP and have persistent severe OSA are appropriate candidates for tracheostomy. Tracheostomy allows the obstructive airway to be bypassed. A significant degree of counseling is involved in preparation for surgery due to the life altering changes a family will experience managing a child with a tracheostomy. Drawbacks of tracheotomy include further impairment to already rudimentary communication skills, increased need for specialized and institutionalized care, and decreased quality of life (83). Complications associated with tracheostomy include subglottic stenosis, airway obstruction, granulation tissue, bleeding, and death (84).

## Anesthetic Implications

Pre-operative sedation should be used with caution in patients with severe OSAs. During induction of anesthesia, there is high risk for airway obstruction, desaturation, and laryngospasm (85). Patients with severe OSAs have an abnormal ventilator response to carbon dioxide and are likely to have greater respiratory depression in response to sedatives, narcotics, and general anesthetics, and can have significant delay in the return to spontaneous ventilation and emergence from general anesthesia (85). The presence of trace volatile anesthetics will further reduce what may be pre-existing abnormal ventilatory drive and potentiate airway obstruction due to reduced function of the genioglossus and other airway muscles (86). After extubation patients are at risk for post-extubation obstruction, laryngospasm, desaturation, pulmonary edema, and respiratory arrest (87).

Children who should be observed after surgery include age under three and those with severe OSAs (apnea–hypopnea index of 10 or more obstructive events/hour, oxygen saturation nadir less than 80%, or both) (32, 88). Monitoring in an intensive care unit may be warranted in a child with very severe OSAs, medical comorbidities that cannot be managed on the floor, and those who demonstrate significant airway obstruction and desaturation in the initial post-operative period that requires interventions beyond repositioning and/or oxygen supplementation (15). Post-operative respiratory compromise has been reported to occur in 16–27% of children with OSAs (69, 88, 89). Despite an increased risk for complications, it is advantageous to extubate patients immediately after surgery if criteria are met. Children who remained electively intubated had a higher complication rate (47%) than those who did not (2%) (90).

## Conclusion

The evaluation of the patient of UAO includes a thorough history and physical examination. Risk factors including obesity, Down syndrome, craniofacial disorders, or neurologically impaired may indicate the need for pre-operative PSG. Adenotonsillectomy is effective in the majority of children, but increasing rates of residual OSAs are seen, particularly in obese patients and those with Down syndrome. For patients with residual OSAs, multiple level surgery may be undertaken after evaluation with flexible endoscopy and/or imaging studies to identify the levels of obstruction. Children with OSA are at increased risk for peri-operative complications and should be monitored accordingly post-operatively.

## Conflict of Interest Statement

The Review Editor Michael E. McCormick declares that, despite being affiliated to the same institution as author Cecille G. Sulman, the review process was handled objectively and no conflict of interest exists. The author declares that the research was conducted in the absence of any commercial or financial relationships that could be construed as a potential conflict of interest.
